# Effects of Super Nutritional Hepatic Copper Accumulation on Hepatocyte Health and Oxidative Stress in Dairy Cows

**DOI:** 10.1155/2019/3642954

**Published:** 2019-05-05

**Authors:** Jaimie M. Strickland, Doug Lyman, Lorraine M. Sordillo, Thomas H. Herdt, John P. Buchweitz

**Affiliations:** ^1^Department of Large Animal Clinical Sciences, College of Veterinary Medicine, Michigan State University, 784 Wilson Rd, East Lansing, MI 48823, USA; ^2^Department of Pathobiology and Diagnostic Investigation, College of Veterinary Medicine, Michigan State University, 784 Wilson Rd, East Lansing, MI 48823, USA; ^3^Veterinary Diagnostic Laboratory, College of Veterinary Medicine, University of Wisconsin, 445 Easterday Ln., Madison, WI 53706, USA

## Abstract

Concerns regarding excessive hepatic copper concentrations in dairy cows have increased. The objective of this study was to determine the association of hepatic copper concentrations with evidence of liver disease. Blood and liver samples were collected at the time of slaughter in cull dairy cows (n=100). Liver samples were analyzed for copper using inductively coupled plasma mass spectrometry and crude fat using liquid-liquid extraction and gravimetry. Serum samples were analyzed for glutamate dehydrogenase, *γ*-glutamyltransferase, sorbitol dehydrogenase, aspartate aminotransferase activities, and bile acid concentrations. Liver samples were examined histologically for inflammation, fibrosis, and rhodanine staining. Animals were stratified by hepatic copper concentration and samples in the highest and lowest quintiles (Q5 and Q1) were evaluated for oxidative stress. Systemic indices of oxidative stress included serum reactive oxygen and nitrogen species (RONS) and total antioxidant potential (AOP). Tissue-level oxidative stress was assessed by immunohistochemistry using 4-hydroxynonenal (4HNE) and 3-nitrotyrosine (3NIT) stains to score the relative abundance and distribution of oxidized lipid and protein products, respectively. Mean hepatic copper concentration was 496.83 *μ*g/g and median 469.72 *μ*g/g and ranged from 70.56 to 1264.27 *μ*g/g dry tissue. No association was found between hepatic copper concentrations and clinicopathological or histological evidence of hepatic damage or dysfunction. There was a significant increase in the amount of IHC staining of 4HNE and 3NIT in Q5 compared with Q1. Moreover, the IHC staining mirrored the distribution of the copper-specific stain rhodanine. These results demonstrate that cows with elevated hepatic copper concentrations had no evidence of active liver disease but had increased hepatic oxidative stress.

## 1. Introduction

Copper is an essential cofactor in hundreds of enzymatic reactions and is a necessary component of the diet of all species [1]. The digestion of copper in the ruminant animal is complex due to the potential antagonistic interactions of molybdenum, sulfur, and iron in the rumen [2]. Therefore, copper is often supplemented in the diets of dairy cattle and oversupplementation can occur [3]. Copper is sequestered in liver and thus hepatic copper concentration is the best measure for total body copper status. Serum copper concentrations do not adequately represent hepatic copper concentrations unless the animal has extremely deficient or toxic concentrations [1]. Hepatic copper concentrations are considered to be super nutritional when concentrations exceed that which is needed to maintain health. The range of sufficient hepatic reserves is ill-defined, but the expected upper limit is thought to be between 300 and 500 *μ*g/g dry tissue [1, 4]. Due to its high redox potential, excessive hepatic copper could cause oxidative damage to hepatocellular lipids, proteins, and DNA. This can lead to organelle dysfunction and apoptosis [5]. Abnormal hepatic copper accumulation leading to liver disease occurs in sheep, dogs, and humans [6–8]. If left untreated, excessive hepatic copper accumulation in these species can lead to hepatitis, cirrhosis, hepatic necrosis, and death [9, 10]. Excessive hepatic copper accumulation in dairy cattle has been of growing concern in recent years [11–14]. Despite these accounts, the impact of elevated hepatic copper concentrations on bovine health, short of fulminant toxicosis, is not well understood.

Damaged hepatocytes release hepatocellular enzymes into circulation where they can be measured in peripheral blood by routine clinicopathological tests. Serum activities of these enzymes are therefore commonly used as biomarkers for hepatopathies. Clinicopathological analysis for serum hepatocellular leakage enzyme activities is a critical feature in clinical diagnosis of many hepatopathies in a variety of species, including copper accumulation hepatopathies in dogs, sheep, and man [6, 15, 16]. However, several of the serum enzymes activities that may be elevated in liver disease are not liver-specific and may originate from other cells in the body. For this reason, it is common to measure the activity of multiple serum liver enzymes in one sample to increase the specificity of diagnosis for liver damage. Common liver enzymes measured in dairy cattle include glutamate dehydrogenase (GLDH), *γ*-glutamyltransferase (GGT), sorbitol dehydrogenase (SDH), and aspartate aminotransferase (AST).

When copper intake exceeds liver storage and the export capabilities, then free copper is more likely to occur. Free copper has prooxidant potential and in excess can lead to increased oxidative stress through the Fenton reaction [17]. Oxidative stress occurs when reactive oxygen and nitrogen species (RONS) overwhelm the organism's total antioxidant potential (AOP) resulting in damage to cells. In dairy cattle, the oxidant stress index (Osi) which is the ratio serum RONS/AOP has been established in order to associate oxidative status with disease risk [18]. Lipids are particularly vulnerable to oxidative stress, but proteins and DNA are also susceptible. This can lead to organelle dysfunction and apoptosis [5]. Indeed, oxidative stress has been linked to dysfunctional immune and inflammatory responses in dairy cows [19]. Oxidative stress in relation to hepatic copper concentrations in dairy cattle has only recently been investigated [20, 21].

The prooxidant characteristics of metallic copper may be a link between high hepatic copper concentrations and clinical disease due to increased risk for oxidative stress. Our group has demonstrated that high hepatic copper concentrations occur frequently in dairy cows, but the clinical significance of hepatic copper concentrations in this range is unknown [22]. The aim of this study was to determine if an association exists between super nutritional hepatic copper concentrations and evidence of hepatocellular damage in dairy cattle by utilizing multiple measurements of liver damage including serum liver enzyme activities, serum bile acid concentrations, and systemic and hepatic oxidative stress. If such an association is verified, it will suggest elevated but subtoxic hepatic copper concentrations can result in subclinical liver damage in dairy cows.

## 2. Methods

Paired liver and blood samples (n=100) from cull dairy cows were collected at the time of slaughter at a West Michigan abattoir. This study was exempted by the Michigan State University Institutional Animal Care and Use Committee. Blood samples were collected in serum separator tubes (BD Vacutainer Serum Separator) and EDTA Tubes, (Becton, Dickinson and Company, Franklin Lakes, NJ 07417) during exsanguination. Serum was separated from the clot within an hour of collection (9.055.8 xG for 10 min) and stored at 4°C until analysis within 96 hours for liver enzyme activity and bile acid concentration. For RONS and AOP analysis, approximately 1 mL of serum was pipetted into cryo-vial tubes. The vials were flash frozen in liquid nitrogen and then later transferred to a -80°C freezer for storage until analysis.

### 2.1. Mineral Analysis

Hepatic copper concentration was determined on fresh tissue but expressed on a dry tissue basis. Tissues were sectioned (1 gm) and digested overnight in a 95°C oven, using approximately 10 times the dry tissue mass of nitric acid. A separate 2-gm section was dried overnight in a 75°C oven to determine the dry matter fraction and calculate the dried tissue mass. The digested samples were diluted with water to 100 times the tissue mass. Elemental analysis was performed using an Agilent Inductively Coupled Plasma Mass Spectrometer (ICP-MS) (Agilent Technologies Inc., Santa Clara CA 95051) [23]. Briefly, 200 *μ*L of each diluted tissue digest and calibration standard was diluted 20-fold with a solution containing 0.5% EDTA and Triton X-100, 1% ammonium hydroxide, 2.0% propanol, and 5 ppb of scandium, and 7.5 ppb of germanium, rhodium, indium, and bismuth as internal standards. The ICP-MS was tuned to yield a minimum of 7500 cps sensitivity for 1 ppb yttrium (mass 89), less than 1.0% oxide level as determined by the 156/140 mass ratio, and less than 2.0% double charged ions as determined by the 70/140 mass ratio. Elemental concentrations were calibrated using a 5-point linear curve of the analyte-internal standard response ratio. Standards were from Inorganic Ventures (Inorganic Ventures, Christiansburg, VA 24073). NIST Bovine Liver and Mussel standards (National Institute of Standards and Technology, Gaithersburg MD 20899) were used as controls. Copper concentrations were reported on a dry tissue basis.

Animals were stratified based on liver copper concentration and divided into quintiles. Variables associated with oxidative stress were measured in the highest and lowest quintiles (Q5 and Q1). These variables included serum total reactive oxygen and nitrogen species (RONS) and antioxidant potential (AOP).

### 2.2. Clinicopathology

Blood chemistry profiles were performed by Marshfield Laboratories (https://www.marshfieldlabs.org) according to their standard operating procedures. Briefly, all clinicopathological variables were analyzed on the Beckman Coulter AU5800 chemical analyzer (Becker Coulter Diagnostics, Brea, CA 92821). AST, GGT (Becker Coulter Diagnostics, Brea, CA 92821), SDH, GLDH (Catachem Inc., Oxford, CT 06478), and BA (Diazyme Laboratories Inc., Poway, CA 92064) were all analyzed according to the manufacturer's instructions [24–26].

Crude fat analysis was done by solvent extraction using a modified version of a previously published method [27]. Briefly, 1-gm of liver was fully homogenized with 24 ml of hexane:isopropanol (3:2) in 50 ml centrifuge tubes. Next, 12 mL of sodium sulfate was added and samples were mixed for 1 minute each. Samples were then centrifuged (18,514.08 xG for 5 min). The hexane fraction was pipetted off into preweighed glass tubes. The sides of the centrifuged tubes were rinsed with 2 mL of hexane and centrifuged and then the remaining hexane fraction was pipetted into its corresponding glass tube. Once the hexane fully evaporated the glass tubes were weighed again.

### 2.3. Oxidant Status Analysis

AOP was quantified in serum samples as described previously [28]. Briefly, the AOP of a sample was standardized to the reduction capacity of Trolox (synthetic vitamin E analog) in 2, 20-azinobis-3-ethylbenzothiazoline-6-sulfonic acid (ABTS) solution [29]. RONS were quantified in serum as a marker of prooxidant production using a commercially available assay (ROS and RNS assay, Cell Biolabs, San Diego, CA 92126) following the manufacturer's instructions. In brief, free radicals in the sample react with a specific probe that is converted into a fluorescent product. Thus, the fluorescent intensity is proportional to the total RONS content in the sample. Fluorescence of the dichlorofluorescent dye was determined at 480 nm of excitation and 530 nm of emission and a standard dichlorofluorescent dye curve was included to ensure that the dye could be detected at various concentrations. Blank values were subtracted from sample values to eliminate background fluorescence. The reported values represent relative fluorescent units normalized per microliter of sample [30]. Concentrations of RONS and AOP were utilized to calculate the Osi ratio [18].

### 2.4. Immunohistochemistry

Formalin fixed liver sections in Q1 and Q5 (n=40) were stained with immunohistochemistry (IHC) stains for 4-hydroxynonenal (4HNE) and 3-nitrotyrosine (3NIT) at the Department of Veterinary Pathology, Iowa State University. Tissues were cut into 5 micron sections and placed on glass slides. Slides were baked at 57°C for 30 min. Tissues were deparaffinized in xylene and then rehydrated in alcohol. Tissues were then processed with a commercially available Avidin/Biotin blocking kit (Avidin/Biotin Blocking Kit, Cat # 004303, ThermoFischer Scientific, Waltham, MA 02451). In order to inhibit endogenous peroxidase activity, tissues were incubated in 3% hydrogen peroxide for 2, 10 min applications followed by 3 ultrapure water rinses. For the antigen retrieval process slides were placed in a plastic Coplin jar containing Citra pH 6 antigen retrieval buffer. They were then microwaved on high (full power) until the surface was bubbling. The Coplin jar was then moved to a preheated steamer for 20 min. They were then left at room temperature for 20 min and rinsed twice with PBS. Next, the slides were blocked with 90% NGS/(Tris/PBS/BSA buffer) for 20 min. The primary mouse monoclonal antibody was diluted in Tris/PBS/BSA buffer (4-HNE was diluted 1:50 and 3-NIT was dilution 1:100) (Anti-4-Hydroxynonenal Antibody, Abcam, Cambridge, MA 02139 ) (Anti-3-Nitrotyrosine Antibody, Santa Cruz Technology, Dallas, TX 75220) and incubated for 2 hours followed by 2x PBS rinses, 5 min PBS bath, and 2x PBS rinses. Next, samples were processed with dilute Multilink 1:80 in Tris/PBS/BSA buffer that was applied for 15 min followed by 2 PBS rinses, 5 min PBS bath, and 2 more PBS rinses. Then dilute Horseradish Peroxidase-Streptavidin 1:200 in Tris/ PBS/BSA buffer was applied for 15 minutes followed by 2x PBS rinses, 5 minute PBS bath, and 2x PBS rinses. Subsequently, Nova Red stain was applied for 5 min and rinsed 5 times with ultrapure water. Slides were then transferred into 1/4 strength Shandon's hematoxylin for 2 min, rinsed in ultrapure water 3 times, placed in Scott's tap water for 1 min, and then rinsed 3 times in ultrapure water. Slides were then dehydrated with alcohol and Xylene. Coverslip slides were applied with a nonaqueous mounting media (Acrytol Mounting Media, Surgipath, Leica Biosystems, Buffalo Grove, IL 60089) and allowed to dry at room temperature. Formalin fixed liver sections were processed for H&E and rhodanine staining (n=100).

### 2.5. Histopathology Scoring

Rhodanine and IHC stained slides were graded in the same manner on a 0-5 scale. A score of 0 showed no IHC or rhodanine staining. A score of 1 was a section that had minimal granules in less than 33% of centrilobular hepatocytes. A score of 2 indicated there was moderate rhodanine or IHC stained granules in less than 50% of centrilobular hepatocytes. A score of 3 indicated there were moderate to large amounts of rhodanine or IHC stained granules in greater than 50% of the centrilobular hepatocytes. A score of 4 had staining in greater than 75% of zone 3 hepatocytes and a score of 5 demonstrated panlobular staining.

H&E slides were all observed for signs of centrilobular necrosis and were scored on relative amount of inflammation and fibrosis. For inflammation a score of 0 meant that there were no visible inflammatory cells, a score of 1 had a mild inflammatory infiltrate with low numbers of lymphocytes, plasma cells, and histiocytes within the portal region, and sections with a score of 2 had severe inflammation with high numbers of the before-mentioned inflammatory cells and neutrophils within the periportal regions. For fibrosis, a score of zero meant that the section had no fibrosis, a score of 1 meant that the section had mild expansion of the portal areas by dense material fibrosis, and sections with a score of 2 demonstrated severe, bridging fibrosis between portal areas.

### 2.6. Statistics

The associations among continuous variables were examined via an exploratory factor analysis (Proc FACTOR, SAS 9.4). Histograms of the variables were examined visually for their distribution. The serum liver-leakage enzyme activity distributions were skewed to the right in a non-Gaussian pattern, suggesting neither the assumptions of normality nor multivariate normality could be made for these data. Thus, the “unweighted least squares” method of factor extraction, which is robust to nonnormal data distributions [31], was used for factor extraction. An oblique rotation (OBVARIMAX) was applied to the factor analysis. Oblique rotations serve to make factor analysis results easier to interpret and are more realistic than other rotations [32]. Minimum eigenvalues were set at 0.5. A moderate loading was declared at 0.4 to 0.7 and a strong loading was declared at >0.7, with regard to its absolute value. Although loadings less than 0.4 are not necessarily unimportant when evaluating factor analyses, those loadings were not considered for interpretation in this study [33, 34]. The matrix of individual Pearson product-moment correlation coefficients was generated by Proc CORR, SAS 9.4.

Differences in copper concentrations across the histologically determined score categories for inflammation, fibrosis, necrosis, and rhodanine staining were evaluated by the Kruskal-Wallis test. Differences in mean values for serum RONS, AOP, and IHC scores in Q1 and Q5 were assessed by the Mann-Whitney* U* test (GraphPad Prism, version 6.04). The association between liver copper concentration and rhodanine score was determined by Pearson correlation coefficient. Significance was declared at P < 0.05.

## 3. Results

### 3.1. Mineral Analysis

The mean hepatic copper concentration was 496.83 *μ*g/g and median was 469.72 *μ*g/g, and the concentrations ranged from 70.56 to 1264.27 *μ*g/g (reference range: 40-650 *μ*g/g) (Michigan State University Veterinary Diagnostic Laboratory, Lansing, Michigan, 49810). These results were similar to a previous abattoir study of cull dairy cows performed by our group in that the range of hepatic copper concentrations substantially exceed those necessary for nutritional adequacy [4]. The mean hepatic copper concentrations for Q1 and Q5 were 215.19 *μ*g/g and 835.36 *μ*g/g, respectively.

### 3.2. Clinicopathology

**Figure 1:**
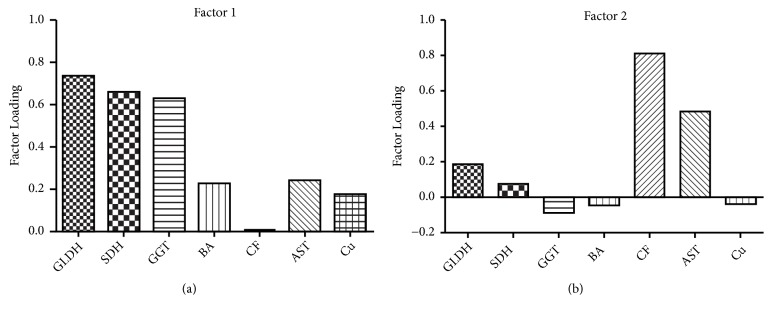
Factor analysis of liver-leakage enzymes, bile acids, and hepatic crude fat with an oblique rotation represented in graphic form (n=100). A moderate loading was considered to be 0.4-0.7 and a strong loading was considered >0.7-1.0, in absolute values. The latent variables are represented as (a) hepatocyte health factor and (b) hepatic lipidosis factor.

Serum liver-leakage enzyme activity, as well as serum bile acid and hepatic crude fat concentration results, is shown in Table 1.  Table 2 is a correlation matrix of the continuous variables used in the factor analysis. The factor analysis resulted in 2 factors with eigenvalues in excess of 0.5. Factor 1 included strong positive loadings for serum GLDH and moderate positive loadings for serum SDH and GGT. As these variables are associated with hepatocyte and bile duct epithelium health and function we assigned to this factor the description “hepatic health” factor (Figure 1(a)). Liver copper concentration was not associated with factor 1 having a very low loading. Factor 2 had a strong positive loading for liver crude fat percentage and a moderate loading for serum AST. We assigned to this factor the description “hepatic lipidosis” factor (Figure 1(b)). The loading for hepatic copper concentration on factor 2 was -0.38, slightly outside our declared value range (±0.4) for “moderate.” It is, however, important to note the hepatic crude fat concentration loading on factor 2 was positive and strong (0.8), indicating an inverse relationship between hepatic copper and crude fat concentrations. Serum BA concentration did not have a significant loading in either factor.

There was no difference in serum RONS or the Osi ratio between Q1 and Q5. There was, however, a significant increase in serum AOP in Q5 compared to Q1. This may suggest a compensatory response in the redox status of the cows in Q5 (Table 3).

### 3.3. Immunohistochemistry

**Figure 2:**
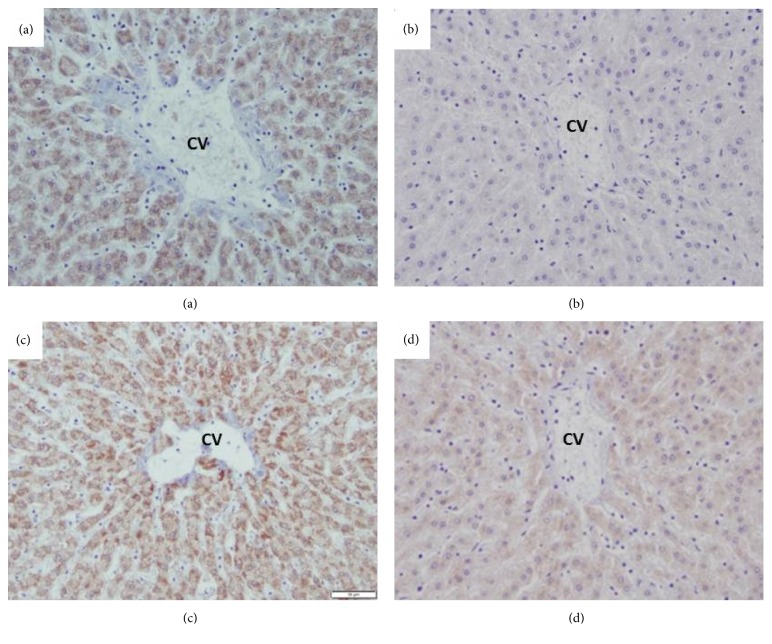
The 3-nitrotyrosine (3NIT (a, b)) and 4-hydroxynonenal (4HNE (c, d)) stained slides for two separate samples centered on the zone 3 hepatocytes where copper accumulates first in the bovine liver. Central vein (CV) is labeled and slides are magnified at 20x. Cow 1 (a, c) had a hepatic copper concentration of 1264.27 *μ*g/g. Cow 2 (b, d) had a hepatic copper concentration of 173.24 *μ*g/g (ref. range: 40-650 *μ*g/g).

IHC staining scores in Q5 were significantly higher than Q1 for both 4HNE and 3NIT (p = 0.0003 and p = 0.0058, respectively) (Table 4). Interestingly, the IHC stain pattern mirrored that of rhodanine in that it originated from the centrilobular area suggesting that oxidative stress was associated with lysosomal copper stores (Figure 2).

### 3.4. Histological Scoring

**Figure 3:**
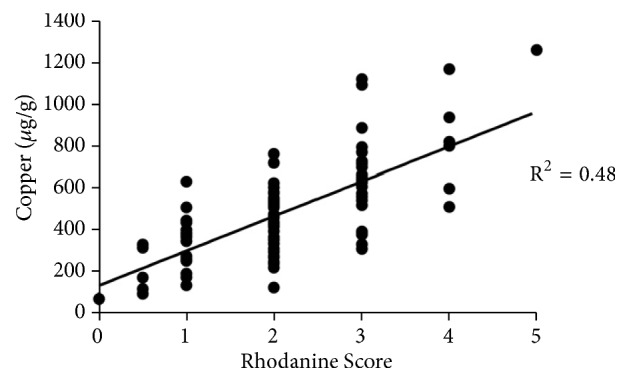
Correlation of quantitative copper concentrations (*μ*g/g) and rhodanine scores (n=100).

Hepatic necrosis is a frequent consequence of copper intoxication; however H&E staining revealed no evidence of hepatic necrosis in any of the animals studied. In addition, there was minimal evidence of inflammation or fibrosis. Indeed, there were only 2 cows with fibrosis scores greater than 0 so Kruskal-Wallis could not be utilized. There was no correlation between hepatic copper concentrations and inflammation (P = 0.937). There was a significant positive relationship (r^2^ = 0.483) between rhodanine score and copper concentration (Figure 3).

## 4. Discussion

In the current study we examined the association among multiple variables related to hepatic injury or dysfunction with hepatic copper concentrations. We expected associations, or factors, to emerge that would represent different aspects, types, or dimensions of hepatic injury or dysfunction. Factor analysis is a powerful means of examining and interpreting associations among multivariate data. The correlation matrix of multiple variables forms the basis of the data for factor analysis. The analysis creates unmeasured variables called factors. The strength of association between each measured variable and each factor is defined by the loading parameter, which can vary between -1 and +1 and is interpreted similarly to a correlation coefficient. A relatively high absolute loading of a measured variable on a given factor indicates the strength of its association, positive or negative, with that factor. Of particular interest in the interpretation of factor analysis is the grouping of measured variables among the factors because these groupings define the biological nature of the factor. The factors are sometimes referred to as latent variables, suggesting they are phenomena of interest but are difficult to measure objectively. Further, and pursuant to our major objective, we wish to see if hepatic copper concentration is associated with one or more of these dimensions of hepatic injury or dysfunction. Such an association within the study population would imply that increasing copper concentration might be a risk factor for subclinical hepatic disease.

Factor 1 was termed the “hepatic health” factor as it had strong loadings for serum GLDH and a moderate loading of serum SDH and GGT, well accepted clinicopathological indicators of bovine hepatic health or integrity [35]. Hepatocyte health is a good example of an unobservable construct within our study that formed through associations with measurable values of liver health [32]. Hepatic copper concentration had a very weak loading on factor 1. In addition, there was no association of microscopic evidence of liver disease with hepatic copper concentrations. Thus, there was no clinicopathological or histological evidence of hepatic disease or compromised function associated with high liver copper concentration. These findings, particularly those associated with serum enzyme interpretation, appear to contrast previous reports in the literature [36–38]. Discrepancies may have resulted from differences in mean hepatic copper concentrations between the present study and contrasting studies, or from use of plasma copper instead of hepatic copper concentrations as a measure of copper status. It has been established that plasma and serum copper concentrations do not adequately represent hepatic stores [1], especially when hepatic copper concentrations are elevated ([39]). In the feeding trial of Minervino et al. [38] serum GGT activity was predictive of liver copper concentration, but not reliably so until the liver copper concentration exceeded 1000 *μ*g/g. Thus, the findings in these contrasting studies suggest the range of liver copper concentrations in the present study, although higher than required for nutritional adequacy for many individuals, was not high enough to consistently induce hepatic disease. This study found no association with elevated hepatic copper concentrations and liver disease as indicated by factor 1. Moreover, we observed serum liver-leakage enzyme activities in this cohort to be generally within the expected reference ranges, irrespective of the broad range of hepatic copper concentrations observed.

Factor 2 was assigned the term “hepatic lipidosis” factor with strong loadings for liver fat percentage and a moderate loading for serum AST. This is consistent with previous investigations that have observed bovine serum AST activity to be more strongly associated with liver fat concentration than the serum activities of other liver enzymes evaluated in this experiment [40–42]. Additionally, factor 2 suggested a weak but apparently inverse relationship between hepatic copper and fat concentrations. Such a relationship is likely due to dilution of protein-rich portions of the hepatocyte by fat, copper being contained in the protein fraction [43]. Similar observations have been found in studies with other species. For example, one study in humans found that patients with nonalcoholic fatty liver disease had 24% less hepatic copper than healthy controls [44].

This study demonstrated that hepatic sections in Q5 showed significantly greater oxidative stress as demonstrated by 4HNE and 3NIT IHC staining than those from Q1 (p = 0.0003 and p = 0.0058, respectively). Moreover, IHC staining distribution mirrored that of rhodanine staining within hepatic lobules (Figure 2). These findings suggest super nutritional, but subtoxic, hepatic copper accumulation does increase risk of local oxidative stress in the liver. 4HNE stains have been used to study the pathogenesis of copper accumulation in Long-Evans Cinnamon (LEC) rats which serve as a model for Wilson's Disease in humans. Positive 4HNE staining occurs in LEC rats prior to detectable inflammation by histopathology [45]. This shows that 4HNE staining may be particularly useful in characterizing subclinical hepatocellular damage caused by copper accumulation in species such as cattle. Other studies have investigated the association between hepatic copper accumulation and oxidative stress in cattle. A Spanish feeding trial demonstrated that copper supplemented calves had increased IHC staining of oxidative stress products (nitrotyrosine and inducible nitric oxide synthase), relative to controls [20]. Another study, which also utilized cull dairy cows, found that many samples with high hepatic copper concentrations also had 4HNE staining that mirrored the distribution of the rhodanine staining [21].

There was a significant increase in AOP in Q5 than Q1. The elevation in AOP may be due to a compensatory increase in thiols following the increase of RONS. This change, however, demonstrates a redox shift in Q5 cows. Compared to results from previous studies, RONS was elevated in both Q1 and Q5 relative to nondiseased cows at dry-off [30]. This suggests that cows in Q1 and Q5 may have been subject to other diseases or sources of RONS unrelated to hepatic copper accumulation. Oxidative stress has been associated with diseases of dairy cattle, with modulation shown to decrease severity [46]. More research is needed to determine if the effects of AOP and RONS seen in this population are directly related to hepatic copper concentrations.

In this study a population of cull cows slaughtered at a small commercial abattoir was used as a convenience sample. Cows are culled for many reasons, but an intense period of involuntary culling occurs early in lactation [47, 48]. In these instances, metabolic disease is a frequent reason for culling. While the clinical history of the animals was unavailable, the general appearance of the animals and the high proportion with high liver fat concentrations suggests many of them were early lactation animals with metabolic problems. This specialized population creates challenges relative to inference of our results to the general population of cows. The metabolic stress of early lactation might have influenced the occurrence of subclinical liver disease (factor 1), exclusive of the potential effects of copper, thus masking an association we had hypothesized. However, the lack of association of hepatic copper with evidence of subclinical liver disease, even in this population that was probably undergoing metabolic stress, suggests copper within the ranges measured here is not associated with increased risk of liver disease. Further studies across a more general cross-section of the dairy cow population, particularly pursuant to the interactions of copper and oxidative stress, are warranted.

## Figures and Tables

**Table 1:** Liver leakage enzymes, BA, and hepatic CF data.

	Mean ± SE	Range	Reference Range
GLDH (U/L)	23.5 ± 1.65	4-80	6-68
SDH (U/L)	19.66 ± 1.03	7-72.6	6.6-37.8
GGT (U/L)	28.07 ± 0.96	13-68	4-41
BA (*μ*mol/L)	28.12 ± 2.70	2-174	0-12
AST (U/L)	91.43 ± 6.01	32-394	48-204
Hepatic Crude Fat (%)	7.96 ± 0.48	4.44-33.11	3-8

Summary of serum liver leakage enzyme activity, bile acid concentration, and hepatic crude fat percentage in cull Holstein dairy cows (n=100).

**Table 2:** Pearson's correlation.

	CF	Cu	GGT	AST	GLDH	BA	SDH
CF	1.0000	-0.3053	-0.0981	0.4009	0.1443	0.0439	0.0627
		0.0020	0.3316	<0.0001	0.1521	0.6643	0.5317

Cu	-0.3053	1.0000	0.1211	-0.1302	-0.0025	0.1719	0.1280
	0.0020		0.2301	0.1966	0.9803	0.0872	0.2043

GGT	-0.0981	0.1211	1.0000	0.1371	0.4377	0.1564	0.4023
	0.3316	0.2301		0.1739	<0.0001	0.1201	<0.0001

AST	0.4009	-0.1302	0.1371	1.0000	0.2348	-0.0236	0.2147
	<0.0001	0.1966	0.1739		0.0187	0.8157	0.0320

GLDH	0.1443	-0.0025	0.4377	0.2348	1.0000	0.2161	0.5091
	0.1521	0.9803	<0.0001	0.0187		0.0308	<0.0001

BA	0.0439	0.1719	0.1564	-0.0236	0.2161	1.0000	0.0603
	0.6643	0.0872	0.1201	0.8157	0.0308		0.5512

SDH	0.0633	0.1280	0.4023	0.2147	0.5091	0.0603	1.0000
	0.5317	0.2043	<0.0001	0.0320	<0.0001	0.5512	

Pearson's correlation of liver leakage enzymes, bile acids, and hepatic crude fat for all of the study samples (n=100). The top number is *ρ*, or the correlation coefficient, and the bottom number is the *p* value for the correlation of the 2 variables.

**Table 3:** Systemic oxidative stress indices.

	Q1	Q5
	Mean ± SE	Mean ± SE
RONS (RFU/*μ*l)	34.03 ± 6.48	39.73 ± 6.52
AOP (TE/*μ*l)	16.05 ± 0.50	18.75 ± 0.51^*∗∗*^
Osi	2.17 ± 0.41	2.18 ± 0.40

Systemic oxidative stress variables for Q1 and Q5 (n=40). Total antioxidant potential (AOP) was higher in Q5 than Q1 (P = 0.0013). No difference was found between Q1 and Q5 for reactive oxygen and nitrogen species (RONS) or oxidant stress index (Osi) (P > 0.05).

**Table 4:** Immunohistochemistry scores.

	Q1	Q5
	Mean ± SE	Mean ± SE
4HNE	0.85 ± 0.19	2.1 ± 0.23^*∗∗∗*^
3NIT	0.2 ± 0.12	1.03 ± 0.25^*∗∗*^

Immunohistochemistry (IHC) scores for Q1 and Q5 (n=40). IHC slides were scored based on the relative amount of staining from 0 for no staining to 5 for pan lobular staining. Four-Hydroxynonenal (4HNE) staining scores were higher in Q5 than Q1 (p < 0.001) and 3-nitrotyrosine (3NIT) staining scores were higher in Q5 than Q1 (p < 0.01).

## Data Availability

The data used to support the findings of this study are available from the corresponding author upon request
